# Novel Advances in Micronutrient Fortification, Detection, and Bioavailability Evaluation

**DOI:** 10.3390/foods15081331

**Published:** 2026-04-11

**Authors:** Chong Xie, Yongbin Han, Yan Xu

**Affiliations:** 1 Whole Grain Food Engineering Research Center, College of Food Science and Technology, Nanjing Agricultural University, Nanjing 210095, China; xiechong@njau.edu.cn; 2 College of Food Science and Technology, Huazhong Agricultural University, Wuhan 430070, China; xuyan@mail.hzau.edu.cn

## 1. Introduction

Micronutrients, including vitamins and minerals, are fundamental to human physiological function, including supporting metabolic processes, immune regulation, and cognitive development across all life stages [1]. Despite global public health efforts, micronutrient deficiencies remain a pervasive public health challenge, affecting billions of people worldwide [2]. Food fortification has been recognized as one of the most cost-effective strategies to combat malnutrition [3]. Besides adding chemically synthesized micronutrients to commonly consumed staple foods during processing, an increasing number of fortification methods have been developed, such as crop breeding [4], natural ingredient blending fortification [5], food-based carrier fortification [6] and in situ fortification by fermentation [7] as well as germination [8]. The continuous advancement of novel technologies has concurrently introduced challenges, encompassing the precise quantification of micronutrients in complex food matrices, the targeted delivery of bioactive compounds to specific organs, the accurate tracking of micronutrient stability during processing, and the integration of fortification strategies into existing food production workflows with no negative impact on quality.

This Special Issue, entitled “Novel Technologies for Micronutrient Fortification, Detection and Bioaccessibility Evaluation”, was launched to address critical research gaps in the field, including the development of efficient and environmentally friendly micronutrient production and delivery systems; the establishment of precise, high-throughput detection methods for trace-level micronutrients in complex food matrices; and the systematic evaluation of the bioaccessibility and real-world health benefits of fortified foods. The collection includes six original research papers spanning analytical method development, natural nutrient source characterization, fortified product formulation, and consumer acceptability assessment, providing multifaceted insights to advance the translation of fortification technologies from laboratory to market.

## 2. Research Topic Overview

Accurate, reliable detection methods are the foundation of both fortification process control and market regulation. Puścion-Jakubik et al. assessed zinc content in 80 commercially available food supplements using atomic absorption spectrometry (Contribution 1). Their findings revealed that only 70% of products contained zinc within the legally permitted range (−20% to +45% of declared values), with 23.75% exceeding the upper limit and 6.25% falling below the minimum requirement. Both deviations pose measurable health risks: excessive zinc intake may interfere with copper absorption and immune function, while insufficient content fails to deliver the expected nutritional benefits. These results underscore the urgent need for continuous market surveillance to ensure consumer safety, particularly in unregulated supplement sectors.

Fan et al. developed a rapid liquid chromatography–tandem mass spectrometry (LC-MS/MS) method capable of quantifying three active forms of vitamin B12 (adenosylcobalamin, methylcobalamin, and hydroxocobalamin) in 8 min (Contribution 2). A key advantage of this method is its ability to exclude pseudo-vitamin B12 interference by targeting unique ion fragments of active B12 analogs. The researchers also identified light exposure as a critical control point, as it can reduce detectable B12 content by approximately 30% during sample preparation. Validation in fermented rice bran and *Propionibacterium freudenreichii* cultures confirmed this method as a robust tool for developing B12-fortified fermented food.

Zhan et al. reported a glycoprotein, named AAG-3, isolated from Auricularia Auricula (Contribution 3). This glycoprotein has a Mw of 18.21 kDa with an O-type glycopeptide bond and typical functional groups of glycoproteins. It maintains stable α-amylase and α-glucosidase inhibitory activity throughout simulated saliva, gastric, and intestinal digestion, while significantly increasing glucose consumption, glycogen synthesis, and key glycolytic enzyme activities in insulin-resistant HepG2 cells. This glycoprotein is a novel candidate for adjuvant type 2 diabetes management and a new natural bioactive ingredient option for functional food development.

Expanding on natural nutrient source characterization, López et al. optimized a microwave-assisted digestion method using response surface methodology to quantify five essential minerals (Fe, Mg, Na, K, and Ca) in nine species of cultivated and wild edible mushrooms from southern Spain and northern Morocco (Contribution 4). The study revealed that mushrooms contribute a high percentage of the recommended daily intake (RDI) for these minerals, with iron content exceeding RDI thresholds in several wild species. These findings position edible mushrooms as a promising, sustainable natural source of minerals for both direct consumption and food supplement formulation.

Technological innovation must be paired with consumer acceptance to deliver public health impact. Podder et al. conducted a sensory evaluation of multiple-micronutrient-fortified (MMF) dehulled red lentils among 150 rural consumers in Bangladesh, a region with high rates of micronutrient deficiency (Contribution 5). The MMF lentils, fortified with eight vitamins and two minerals, showed no significant differences in odor, taste, texture, or overall acceptability compared to unfortified control lentils after cooking, with only minor variation in uncooked appearance ratings. These results demonstrate that staple food fortification can be implemented without compromising sensory properties, a critical prerequisite for large-scale adoption in low-resource settings.

Peñuñuri-Pacheco et al. developed a food-grade oil-in-water nanoemulsion system for vitamin D3 delivery, optimized using a central composite design (Contribution 6). The optimal formulation, containing 9.12% safflower oil, 1.54% pea protein, and 0.4% salt, achieved a vitamin D3 retention rate of 55.1%, with high stability. When incorporated into a functional meat model system, nanoencapsulated vitamin D3 showed significantly higher retention after cooking compared to unencapsulated vitamin D3. This technology addresses a key challenge in vitamin D fortification of processed meats, where thermal processing typically causes significant nutrient degradation.

## 3. Future Perspectives

The studies included in this Special Issue represent meaningful advances in micronutrient research, but several critical gaps remain to be addressed. Future research should prioritize (1) the development of multiplex detection methods capable of simultaneously quantifying multiple micronutrients in complex food matrices; (2) the scale-up of fortification technologies for industrial production, with a focus on reducing environmental footprint and cost; and (3) long-term population-based intervention studies to validate the real-world health impacts of fortified foods. Additionally, cross-disciplinary collaboration between food scientists, public health researchers, and policymakers will be essential to translate these technological innovations into equitable public health solutions, particularly for underserved populations.
